# Cu-doped and 2-propylimidazole-modified nanoceria (CeO_2_@Cu-PrIm) oxidase-like nanozyme for total antioxidant capacity assay of fruits†

**DOI:** 10.1039/d4ra07858f

**Published:** 2025-04-01

**Authors:** Zhendong Fu, Jiahe Qiu, Ping Gong, Danhong Zhang, Liping Wang

**Affiliations:** a Key Laboratory for Molecular Enzymology and Engineering of Ministry of Education, School of Life Sciences, Jilin University Changchun 130012 China wanglp@jlu.edu.cn; b Jilin University Hospital, Jilin University Changchun 130012 China Zhangdanhong@jlu.edu.cn +86-431-8515-5348

## Abstract

The precise and sensitive quantification of total antioxidant capacity (TAC) is indispensable for evaluating the quality of foods rich in antioxidants. In this investigation, a novel nanozyme exhibiting robust oxidase activity was synthesized *via* a sonochemical doping process utilizing copper ions and the modification of 2-propylimidazole onto nanoceria. Subsequently, a CeO2@Cu-PrIm/ox-TMB assay system was successfully formulated, furnishing a practical and highly sensitive analytical tool for TAC determination. The colorimetric sensor exhibited a linear response over a concentration range of 1 μM to 70 μM, accompanied by a limit of detection (LOD) of 1.26 μM under meticulously screened conditions. This assay displayed substantial practical utility for TAC analysis in fruits, attributed to its exceptional accuracy and selectivity. This research endeavor may offer a novel direction for the design of nanozymes and colorimetric biosensors possessing heightened oxidase activity, thereby advancing the field of analytical chemistry and food science.

## Abstract 2

Schematic depicting the reaction system of CeO2@Cu-PrIm/ox-TMB assay for the determination of TAC.

## Graphical abstract

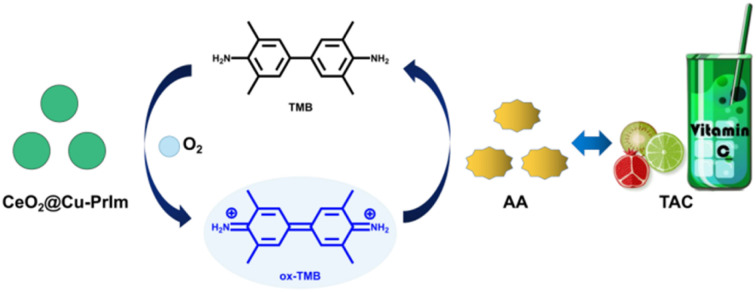

## 1. Introduction

There is accumulating evidence that links reactive oxygen species (ROS) and oxidative damage to a multitude of inflammatory and degenerative diseases, hence, mammals have evolved sophisticated antioxidant mechanisms to efficiently utilize oxygen while minimizing the adverse effects associated with its partially reduced forms.^1^ However, the human body cannot produce most antioxidants and thus they are supplemented by exogenous nutrients rich in antioxidants.^2^ Therefore, antioxidants in exogenous nutrients such as fruits should be quantitatively detected to help people regulate the intake of exogenous nutrients.^3^ Moreover, in contrast to the simple sum of measurable antioxidants, the concept of the integrated parameter of total antioxidant capacity (TAC) represents a comprehensive evaluation of the collective action of all antioxidants.^4^ Various techniques, including hydrogen atom transfer (HAT)-based oxygen radical absorbance capacity (ORAC) assay, electron transfer (ET)-based Folin-Ciocalteu (FC) assay, and ET-based Trolox equivalent antioxidant capacity (TEAC) assay, have been employed for the determination of TAC.^5^ These methods could be combined with other methods, such as spectroscopy,^3,6,7^ chromatography,^8^ and electrochemical techniques^9^ for quantitative analysis. However, these methods are often limited by the shortcomings of expensive and sophisticated instruments, being time-consuming, complicated operations, and the need for experienced experimenters. Hence, the imperative arises to devise an approach that embodies attributes like affordability, time efficiency, and user-friendliness, in order to circumvent certain constraints encountered in the detection of TAC.

The concept of nanozymes has garnered significant attention in the realm of nanomaterials, owing to their high stability, simple preparation at low costs, durability under harsh conditions, and diverse catalytic activities, compared with natural enzymes and artificial enzymes.^10^ Nanozyme is based on transition metal oxides,^11^ precious metals,^12,13^ metal–organic framework,^14^*etc*, simulating catalytic activities of peroxidase,^15^ superoxide dismutase,^16^ and oxidase,^17^*etc.* Furthermore, nanozymes have found extensive application in diverse domains such as *in vitro* sensing, heavy metal ions detection,^18,19^ imaging, therapeutics, waste water treatment,^20–23^ and various other interdisciplinary domains.^24^ Specifically, many nanozymes have been harnessed for the development of biosensors.^25–28^ Nanozymes with oxidase-like activity could avoid the interference of unstable hydrogen peroxide (H_2_O_2_) during the reaction process.^29^ However, only a few studies have assessed TAC through the oxidase activity of nanozymes.^6,30^

**Scheme 1:**
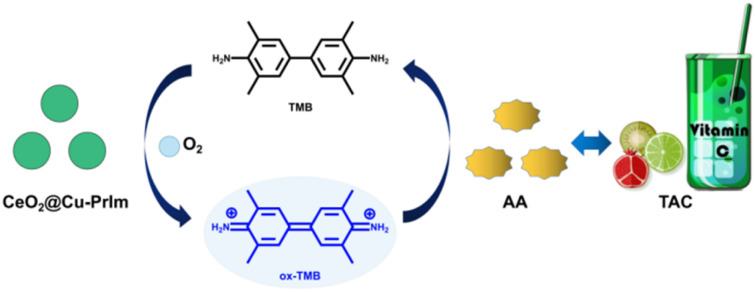
Schematic depicting the reaction system of CeO_2_@Cu-PrIm/ox-TMB assay for the determination of TAC.

Herein, we disign and synthesis a novel nanozyme CeO_2_@Cu-PrIm NPs by bionic approach. The coordination between copper ions and imidazole presence in the active center of natrual laccase and other copper-based oxidoreductases, well cerium doped improved the oxidase activity of the material under acidic conditions. The CeO_2_@Cu-PrIm nanozyme could facilitate rapid electron transfer and could efficiently transform molecular oxygen (O_2_) to the superoxide anion (O_2_˙^−^). This strongly oxidizing intermediate then initiated the catalytic conversion of the colorless compound 3,3,5,5-tetramethylbenzidine (TMB) to the visually distinct blue oxidized TMB (ox-TMB), which exhibited the properties of absorbing light at a wavelength of 652 nm. Incorporating antioxidant substances into the CeO_2_@Cu-PrIm/TMB system enabled the detection of the TAC by monitoring alterations in absorbance.^31^ Taking into consideration these analyses, an uncomplicated colorimetric sensor for the determination of TAC was proposed (Scheme 1). Under the specified screened conditions, the colorimetric sensor exhibited a linear concentration range spanning from 1 μM to 70 μM, coupled with a limit of detection (LOD) of 1.26 μM which could show the great performance of our colorimetric sensor. Furthermore, the DPPH˙ free radicals scavenging experiment was used to substantiate the precision of our proposed TAC assay. In the end, the practical applicability of the reaction system of CeO_2_@Cu-PrIm/ox-TMB assay was demonstrated in the TAC detection of different fruits and health products (*e.g.* kiwi fruits, oranges, tomatoes, orange juice, and vitamin C tablets, *etc.*

## 2. Materials and methods

### 2.1. Materials and reagents

Ceric amine nitrate (Ce(NH_4_)_2_(NO_3_)_6_), copper chloride dihydrate (CuCl_2_·2H_2_O), methanol (CH_3_OH), 2-(*N*-morpholino) ethane sulfonic acid (MES), potassium (KCl), sodium chloride (NaCl), calcium chloride (CaCl_2_), magnesium chloride (MgCl_2_), zinc chloride (ZnCl_2_), glucose (Glu), maltose, fructose (Fru), galactose (Gal), imidazole, 2-methylimidazole, 2-ethyImidazole, 2-propylimidazole, and 2-butylimidazole were procured from Sinopharm Chemical Reagent Co. Ltd (China). Other reagents, including 3,3′,5,5′-tetramethylbenzidine (TMB), *t*-butyl alcohol, 2-furaldehyde (FFA) 1,1-diphenyl-2-picrylhydrazyl (DPPH), and ascorbic acid (AA) were procured from Aladdin Reagent Co. Ltd (China). All these purchased chemical reagents were analytical grade and did not undergo further purification. Kiwi fruits, oranges, tomatoes, and orange juice were sourced from a local store (Changchun, China). Additionally, three types of vitamin tablets were acquired from a local chemist's shop (Changchun, China).

### 2.2. Preparation and characterization of CeO_2_@Cu-PrIm

The synthesis of CeO2@Cu-PrIm *via* the sonochemical method incorporated aspects from our prior research on laccase-mimicking MOFs.^32^ Briefly, 0.2557 g (1.5 mmol) of CuCl_2_·2H_2_O and 1.1016 g (10 mmol) of 2-propylimidazolewere dissolved in a mixture of 5 mL methanol and 15 mL deionized (DI) water. Then, 0.2741 g (0.5 mmol) of Ce(NH_4_)_2_(NO_3_)_6_ and 1.1016 g (10 mmol) of 2-Propylimidazole were also dissolved in a mixture of 5 mL methanol and 15 mL DI water. The two solutions were then slowly mixed and ultrasonicated for 60 minutes. The mixture after ultrasound was centrifuged at 10 000 rpm for 10 minutes and washed thrice with DI water to eliminate the soluble reactants. Finally, the precipitation was freeze-dried for 24 hours. Notably, the synthesis of all nanozymes in this study adhered to a similar procedure, with the sole variation being the doping ratio of copper and cerium metal ions, while maintaining a constant molar amount of 2 mmol of the metal ions.

The morphology and size of the prepared CeO_2_@Cu-PrIm were characterized utilizing a JEM-2200FS field emission electron microscope (TEM). A Nicolet-6700 IR spectrometer (Thermo Scientific, MA, Waltham, USA) was used to detect Fourier transform infrared spectrophotometer (FTIR) spectra at 4000–400 cm^−1^. An X-ray photoelectron spectroscopy (XPS) model ESCALAB250XI (Thermo Scientific, MA, Waltham, USA) with a monochromated Al Kα source was used for XPS. Ultraviolet-visible (UV-vis) spectra were analyzed by UV2501PC (SHIMADZU, Shanghai, China). Empyrean (PANalytical B.V., Netherlands) and Bruker EMXplus (Beijing, China) were used to assess X-ray diffraction (XRD) patterns and Electron spin resonance spectroscopy (ESR), respectively.

### 2.3. Oxidase-like activity of the CeO_2_@Cu-PrIm

The oxidase-like activity of CeO_2_@Cu-PrIm was evaluated using TMB as the chromogenic substrate for the colorimetric assay.^33^ Typically, the TMB solution (100 μL, 10 mM), the CeO_2_@Cu-PrIm solution (100 μL, from 0 to 600 μg mL^−1^), and the MES solution (800 μL, pH 4.0, 30 mM) were mixed thoroughly and then allowed to react at indoor temperatures. The UV-vis spectroscopic measurements were then employed to acquire the absorbance values at 652 nm within the reaction system. Subsequently, the oxidase-like activity was tested as a function of copper and cerium metal ion doping ratio (only Cu, 3 : 1, 1 : 1, 1 : 3, only Ce), nanozyme concentration (0, 10, 20, 30, 40, 50, 60 μg mL^−1^), reaction time (from 0 to 1200 s), or pH (from 2 to 9) change.

### 2.4. Steady-state kinetic experiments

The steady-state kinetic experiments of CeO_2_@Cu-PrIm. Typically, the TMB solution (100 μL, 5 to 50 mM), the CeO_2_@Cu-PrIm solution (100 μL, 500 μg mL^−1^), and the MES solution (800 μL, pH 4.0, 30 mM) were mixed thoroughly and then allowed to incubate at indoor temperatures. The absorbance values at 652 nm of the reaction system were monitored immediately *via* UV-vis spectroscopy measurement. The Michaelis constant (*K*_m_) and maximal reaction velocity (*V*_max_) were determined by fitting the initial reaction velocity with the substrate concentration. The initial reaction velocity of the Michaelis–Menten equation was as follows:^34,35^*ν* = *V*_max_ × [*S*]/(*K*_m_ + [*S*])where *ν* represents the initial reaction velocity, *V*_max_ signifies the maximal reaction velocity, which was characterized by the slope of the concentration–time curve within the initial time, *K*_m_ represents the Michaelis constant, and [*S*] stands for the substrate concentration.

### 2.5. Colorimetric detection of antioxidant

Ascorbate acid (AA) was selected as a representative antioxidant for colorimetric detection.^36^ Typically, the TMB solution (100 μL, 10 mM), CeO_2_@Cu-PrIm solution (100 μL, 500 μg mL^−1^), AA (100 μL, from 0 to 80 μM), and the MES solution (700 μL, pH 4.0, 30 mM) were mixed thoroughly followed by an incubation period of 20 minutes at indoor temperatures. The UV-vis spectroscopic measurements were employed to collect the absorbance values at 652 nm. The range of AA concentration in the reaction that was linear with the absorbance at 652 nm was used as the linear range and provided the data interval for the calculation of the detection limit.

The detection accuracy of the reaction system of CeO_2_@Cu-PrIm/ox-TMB assay was verified using the 1,1-diphenyl-2-picrylhydrazine (DPPH˙) free radicals scavenging experiment.^6,37^ Briefly, a solution containing 100 μM of DPPH˙ was prepared by dissolving it in absolute ethyl alcohol. Subsequently, 100 μL of AA (ranging in concentration from 0 to 70 μM) was promptly introduced to 900 μL of the aforementioned solution. The mixture was then left to incubate in darkness at room temperature for a duration of 30 minutes. Subsequently, the UV-vis spectroscopic measurements were employed to acquire the absorbance values at 517 nm within the reaction system. The detection of antioxidants was carried out through the linear relationship between the ratio of DPPH˙ free radicals scavenged and the concentration of AA.

### 2.6. Detection for TAC of real samples

For the TAC detection using the reaction system of CeO_2_@Cu-PrIm/ox-TMB assay, real samples (vitamin C tablets, kiwi fruit, orange, tomato, and orange juice) were utilized instead of AA, thus assessing the practical applicability of the method. The concentrations of these real samples were adjusted to fall within the linear range of the AA assay. Typically, the TMB solution (100 μL, 10 mM), the sample solution (100 μL), the CeO_2_@Cu-PrIm solution (100 μL, 500 μg mL^−1^), and the MES solution (700 μL, 30 mM, pH 4.0) were mixed thoroughly followed by an incubation period of 20 minutes at indoor temperatures. Subsequently, the absorbance values of the resultant mixture were measured at 652 nm and were incorporated into the standard curve of the AA assay and converted to millimolar equivalents of AA. Ultimately, the determination of TAC content in the samples was achieved by multiplying the millimolar equivalents of AA by a specific dilution ratio, employing the millimolar equivalent of AA per liter (mmol AA per L) as the unit for expression.

## 3. Results and discussion

### 3.1 Characterizations of CeO_2_@Cu-PrIm

**Fig. 1:**
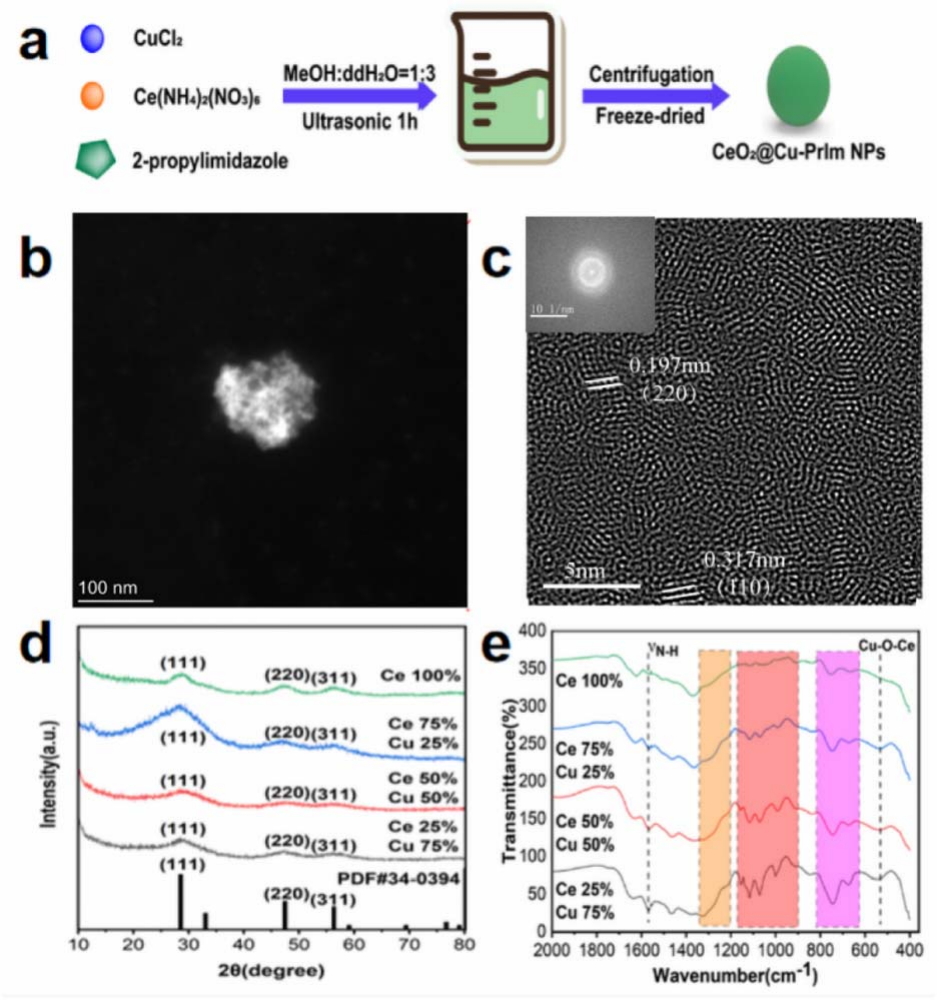
Preparation and characterization of CeO_2_@Cu-PrIm. (a) Schematic depicting the synthesis process. (b) TEM image of CeO_2_@Cu-PrIm. (c) HRTEM image of CeO_2_@Cu-PrIm. (d) XRD pattern. (e) FT-IR spectral analysis. (d) and (e) Contained samples synthesized using different copper and cerium metal ion doping ratios and the grey line represented CeO_2_@Cu-PrIm.

The synthesis of CeO_2_@Cu-PrIm utilizing the sonochemical method as illustrated in Fig. 1a. Initially, a mixed solution containing copper ions and 2-propylimidazole served as the precursor for the metal–organic framework^.^^38,39^ Secondly, CeO_2_ was confined within the Cu-PrIm NPs during crystallization. The CeO_2_@Cu-PrIm nanozymes aggregated into larger particles, which were formed by the coagulation of fine crystals.^39^ The morphology and size of the CeO_2_@Cu-PrIm were obtained through TEM and HRTEM. The former image suggested a cubic fluorite structure of CeO_2_@Cu-PrIm (Fig. 1b), and the latter image showed that the crystallographic spacing of the (111) crystal plane of CeO_2_ increased from 0.3123 nm to 0.317 nm and the (220) crystal plane raised from 0.191 nm to 0.197 nm after doping with copper and 2-Propylimidazole (Fig. 1c).^40^

To further explore the structure and functional groups of the as-prepared CeO_2_@Cu-PrIm, XRD pattern, and FT-IR spectroscopy were conducted. The diffraction peaks of the XRD pattern were consistent with those of CeO_2_ (Fig. 1d). The obvious differences in the XRD pattern could be attributed to the possible differences between the structure of CeO_2_@Cu-PrIm and CeO_2_ caused by the addition of copper ions and 2-propylimidazole.^40,41^ The functional groups of the as-prepared CeO_2_@Cu-PrIm nanozyme were identified by the FT-IR spectra characterization. The peak at 1569 cm^−1^ was attributed to the N–H stretching vibration of the imidazole ring (Fig. 1e). The intricate and intense bands between the 1350–1500 cm^−1^ spectral range were associated with the stretching of the entire ring. The in-plane bending of the imidazole ring was associated with the band between the spectral range 900 and 1350 cm^−1^, while the out-of-plane bending vibration of the imidazole ring was associated with the band between the spectral range 674 and 746 cm^−1^.^32,42^ The peak at 540 cm^−1^ was attributed to a new active site Cu–O–Ce bonds that represents alterations in the original electron distribution and catalytic performance.^43^

**Fig. 2:**
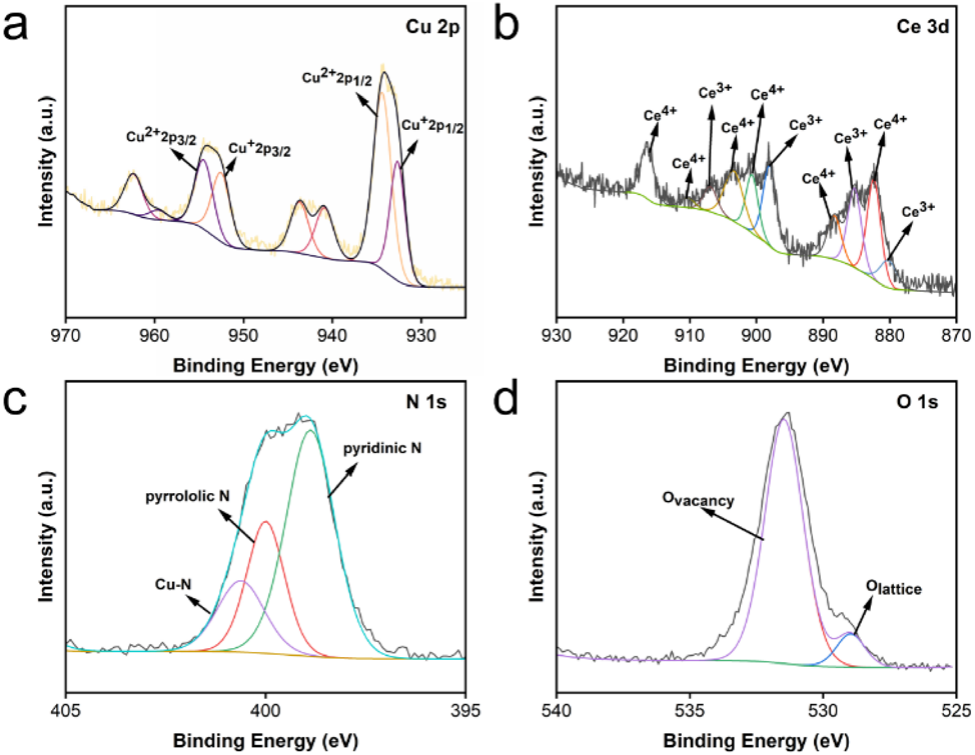
The XPS spectra of CeO_2_@Cu-PrIm. (a) Cu 2p. (b) Ce. 3d (c) N 1s. (d) O 1s.

In addition, the surface elements and corresponding valence states of the prepared CeO_2_@Cu-PrIm were analyzed *via* XPS. The Cu 2p, Ce 3d, O 1s, N 1s, and C 1s peaks in the spectrum occurred at 930 eV, 900 eV, 532 eV, 400 eV, and 286 eV, respectively (Fig. S1†). Based on the high-resolution Cu 2p spectrum, the four peaks of 934.34 eV, 932.66 eV, 954.47 eV, and 952.52 eV were attributed to the Cu^2+^ 2p_1/2_ peak, Cu^+^ 2p_1/2_ peak, Cu^2+^ 2p_3/2_ peak and Cu^+^ 2p_3/2_ peak respectively (Fig. 2a). In addition, the bending energy of Cu 2p was attributed to the transition metal-induced oscillatory satellite peaks.^44^ Quite close characteristic peaks were found by reference Cerium. The appearance of peaks at 880.24 eV, 885.3 eV, 898.0 eV, and 906.94 eV for Ce^3+^, while other peaks at 882.39 eV, 888.32 eV, 900.67 eV, 903.39 eV, 910.4 eV, and 916.39 eV were attributed to Ce^4+^ (Fig. 2b).^45^ The N element is displayed (Fig. 2c). The peaks at 398.8 eV and 400 eV were the pyridinic N and pyrrololic N of the propylimidazole, and the peak at 400.6 eV might be due to the corresponding copper ions and N. The O element was also shown (Fig. 2d). The peak at 531.47 eV was the O vacancy, and the peak at 528.97 eV was the CeO_2_ crystal lattice. The change in the valence state of cerium might be due to copper doping breaking the CeO_2_ crystal lattice, and oxygen vacancies were generated at the same time.^32^ The above results indicated that Cu-doped and propylimidazole-modified nanoceria (CeO_2_@Cu-PrIm) with potential oxidase-like mimetic activity were successfully synthesized.

Subsequently, the batch stability, storage stability, and reusability of the oxidase-like catalytic of CeO_2_@Cu-PrIm were evaluated. The CeO_2_@Cu-PrIm exhibited very similar catalytic activities among five different batches (Fig. S2a†), and there was no remarkable change in catalytic activity during storage for 30 days (Fig. S2b†). Furthermore, there was no significant disparity in catalytic activity between the pre-centrifugation and post-centrifugation states (Fig. S3†), suggesting that the catalytic activity was not significantly contributed by dissolved metal ions. These results proved that the oxidase-like activity of CeO_2_@Cu-PrIm had great attributes with batch stability, storage stability, and reusability, which provided an important basis for the advancement of stable and highly responsive colorimetric sensors.

### 3.2. Oxidase-like activity and screening catalytic conditions of CeO_2_@Cu-PrIm

**Fig. 3:**
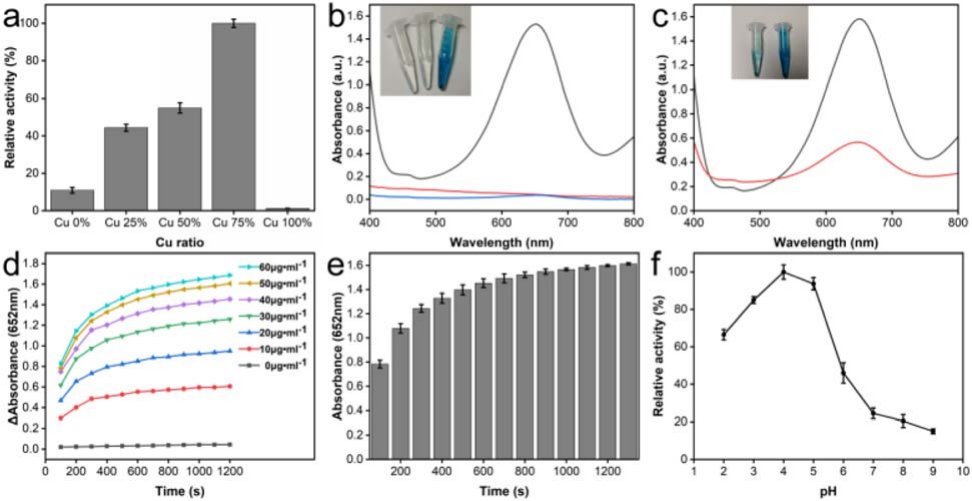
Oxidase-like activities and optimal catalytic conditions of CeO_2_@Cu-PrIm. (a)The oxidase-like catalytic activity of CeO_2_@Cu-PrIm was found to be influenced by the metal doping ratio and pH when using 1 mM TMB as the substrate. (b) UV-vis absorbance spectra demonstrated the catalytic impact of CeO_2_@Cu-PrIm on 10 mM TMB, with a reaction time of 20 minutes. The catalytic oxidation of 100 μL solutions of 10 mM TMB by the as-prepared CeO_2_@Cu-PrIm in a pH 4.0 MES buffer was depicted in the typical photographs accompanying visuals (from left to right:  0 mM TMB with CeO_2_@Cu-PrIm, 1 mM TMB without CeO_2_@Cu-PrIm, 1 mM TMB with CeO_2_@Cu-PrIm). (c) UV-vis absorption spectra of CeO_2_@Cu-PrIm in N_2_-saturated, and air-saturated MES acid buffer. The catalytic oxidation was depicted in the typical photographs accompanying visuals (from left to right:  N_2_-saturated, air-saturated). The catalytic activity of CeO_2_@Cu-PrIm with 1 mM TMB was influenced by the (d) nanozyme concentration (e) reaction time and (f) pH.

To evaluate the oxidase-like activity and identify optimal catalytic conditions for CeO_2_@Cu-PrIm, the synthesized material was assessed using TMB as a chromogenic substrate. The absorbance of theCeO_2_@Cu-PrIm/ox-TMB reaction system at 652 nm was observed to vary based on the metal doping ratio and the pH gradient (Fig. 3a). Notably, within the pH range of 4 to 7, the catalytic activity of the nanozymes increased as the pH decreased, with the exception of nanozymes that did not contain cerium. Among these, the material with a metal doping ratio of 3 : 1 exhibited the highest catalytic activity at a pH of 4, thereby establishing the metal doping ratio for subsequent use. The absorption peak of the CeO2@Cu-PrIm/TMB system was identified at 652 nm (Fig. 3b). However, this absorption peak was absent in control groups lacking either TMB or CeO2@Cu-PrIm, indicating the specificity of the reaction. Furthermore, previous studies have suggested a correlation between the catalytic efficiency of oxidases and the dissolved oxygen levels within the system, suggesting that this factor may also play a role in the catalytic performance of CeO_2_@Cu-PrIm.^46^ Therefore, the catalytic mechanism of CeO_2_@Cu-PrIm was investigated through a comparative analysis of the intensity of absorption peaks at specific wavelengths in nitrogen (N_2_) and air atmospheres, respectively (Fig. 3c). The results indicated that the introduction of N_2_ had the capacity to inhibit the catalytic activity. The above findings confirmed the oxidase-like activity of CeO_2_@Cu-PrIm, thereby underscoring its potential as an effective mimic of oxidase.^47^

The impact of the concentration of CeO_2_@Cu-PrIm on the oxidase-like activity was assessed across the spectra from 0 to 60 μg mL^−1^ (Fig. 3d). Moreover, an escalation in oxidase-like activities was clearly observed in relation to the concentration of CeO_2_@Cu-PrIm was shown (Fig. S4†). Due to the gradual slowing of the increase in oxidase-like activity caused by the increase in concentration, the concentration of 50 μg mL^−1^ was used as the optimal concentration of CeO_2_@Cu-PrIm in the subsequent catalytic system. In order to obtain great detection stability, the reaction time was selected as 20 min (Fig. 3e). The high catalytic activity of CeO_2_@Cu-PrIm was obtained in the pH within the range of 2.0 to 5.0, with the optimal pH being 4.0 (Fig. 3f). According to the screening results of the above experimental conditions, some experimental conditions were unified in the subsequent experiments, the concentration of CeO_2_@Cu-PrIm was 50 μg mL^−1^, the pH of the MES solution was 4.0, and the reaction time was standardized to 20 minutes.

### 3.3. The steady-state kinetic assays of CeO_2_@Cu-PrIm

**Fig. 4:**
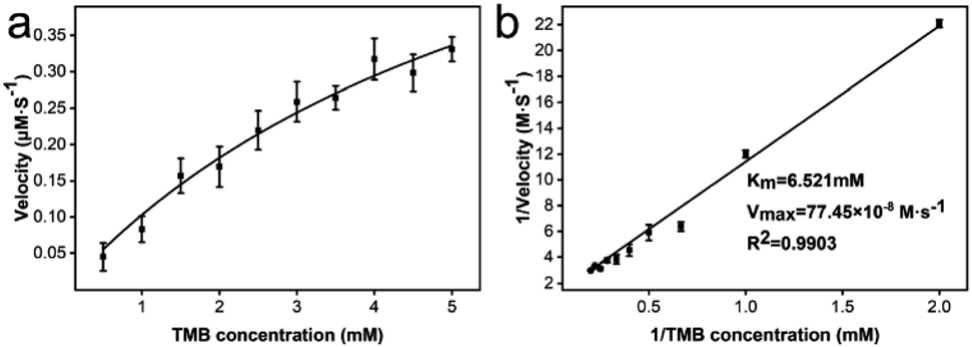
The steady-state kinetic assays of CeO_2_@Cu-PrIm. (a) The steady-state kinetic assays across a range of TMB concentrations (from 0.5 to 5 mM) in a pH 4.0 MES solution. (b) The corresponding Lineweaver–Burk plot of CeO_2_@Cu-PrIm.

To obtain kinetic constants of CeO_2_@Cu-PrIm, the steady-state kinetic assays were conducted under the specified screened conditions. Typical kinetic constants include the Michaelis constant (*K*_m_) and maximum reaction velocity (*V*_max_). The typical Michaelis–Menten curve for CeO_2_@Cu-PrIm was obtained by fitting the substrate concentration TMB (0.5 to 5 mM) from the results of steady-state kinetic assays with the corresponding initial reaction velocity data (Fig. 4a). The corresponding Lineweaver–Burk plot was also obtained using the reciprocal of the data, where the slope and *y*-intercept facilitated a direct calculation of the *K*_m_ and *V*_max_ (Fig. 4b).

The *K*_m_ and *V*_max_ of the oxidase-like activity of CeO_2_@Cu-PrIm were 6.521 mM and 77.45 × 10^−8^ M s^−1^, respectively (Table S1†). Interestingly, despite the higher *K*_m_ value, the *V*_max_ value of CeO_2_@Cu-PrIm was higher than the previously reported nanoceria nanozymes. This demonstrated the advantage of our synthetic method in the rational design of nanoceria with high oxidase properties (Table S2†).

### 3.4. Catalysis mechanism of oxidase-like activity exhibited by CeO_2_@Cu-PrIm

**Fig. 5:**
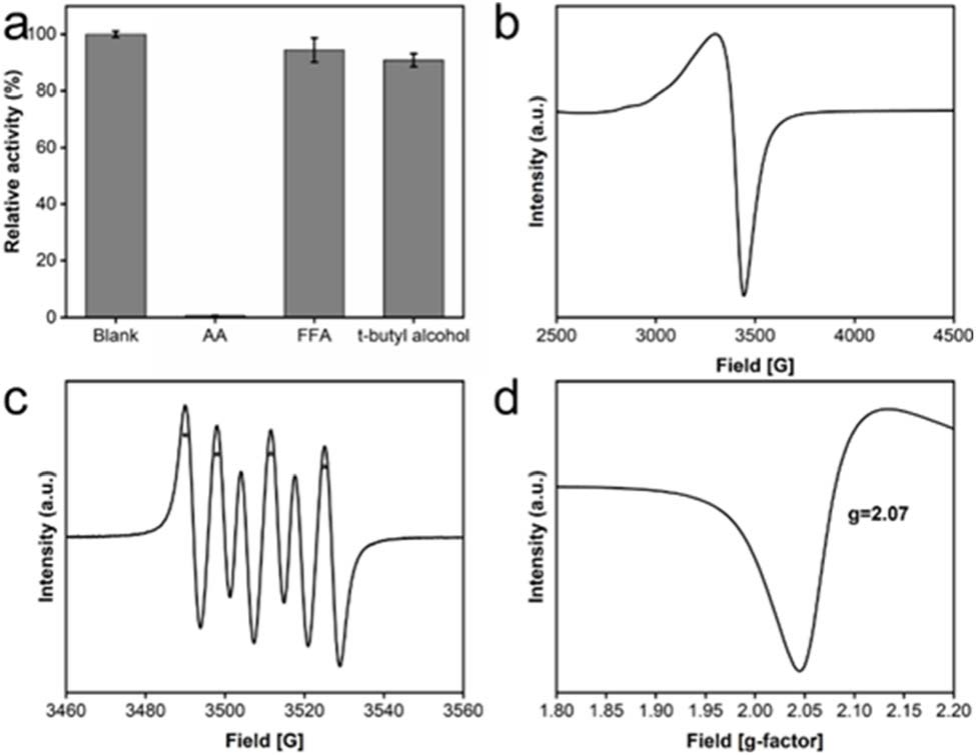
Determination of possible catalytic mechanisms. (a) Free-radical quencher experiments of AA, FFA, and *t*-butyl alcohol. (b) ESR peak patterns of TEMPO/O_2_˙^–^. (c) and (d) ESR spectrum of O vacancy.

To elucidate the catalytic mechanism underlying the observed oxidase-like activity exhibited by CeO_2_@Cu-PrIm, free-radical quencher experiments and ESR spectroscopy were performed to confirm the possible important free radicals in the catalytic reaction. The free radical quenchers introduced into the detection system in the free radical quencher experiment include *t*-butyl alcohol used to quench ˙OH, 2-Furaldehyde (FFA) used to quench ^1^O_2_, and ascorbic acid used to quench ˙OH and O_2_˙^−^, respectively.^48^ The results showed that the relative activity of the quenched ˙OH or ^1^O_2_ reaction system was not significantly affected, while the activity of the quenched O_2_˙^−^ reaction system experienced a significant reduction. This observation underscored the pivotal role played by the intermediate product O_2_˙^−^ free radicals in the catalytic (Fig. 5a). ESR spectroscopy experiments were used to further confirm the generation of O_2_˙^−^ radicals. The characteristic ESR peak patterns of TEMPO/O_2_˙^−^ adduct, characterized by an intensity ratio of 1 : 1:1 : 1, provided evidence that the O_2_˙^−^ radicals were generated in CeO_2_@Cu-PrIm methanol solution (Fig. 5b). Oxygen vacancies could provide hole electrons to activate oxygen to superoxide anion, so the peculiar patterns of O vacancies were illustrated (Fig. 5c and d). Oxygen vacancies could provide hole electrons to activate oxygen to superoxide anion.

Binding sites of oxygen for the catalytic process could be provided by the O vacancies. The reaction was as follows:O_2_ + O_v_^2−^ → O_2_^˙−^ + O_v_^−^

In some previous studies on Ce-based materials, the possible mechanism of the oxidase-like activity was attributed to the Ce^3+^/Ce^4+^ system with the attitude of spontaneous cycling existing in Ce-based materials.^31,49^ In our work, the oxidase-like activity observed for CeO_2_@Cu-PrIm might also be attributed to the Ce^3+^/Ce^4+^ system retained by Ce-based materials. During the catalytic process, TMB was oxidized by Ce^4+^ and transformed into ox-TMB. The imbalance caused by the increase of Ce^3+^ and the decrease of Ce^4+^ in the Ce^3+^/Ce^4+^ system would be restored by the spontaneous cycle between Ce^3+^ and Ce^4+^. The Cu^+^/Cu^2+^ system might play a synergistic role in this process (Fig. S1†).

In this study, the findings revealed that the oxidase-like activity of CeO_2_ was bolstered through the incorporation of copper doping and the modification with 2-propylimidazole. The underlying mechanism behind this enhanced catalytic activity can be attributed to two primary factors. Firstly, Cu^2+^, serving as a low-valence dopant, substituted Ce^4+^ in the lattice, resulting in the creation of electron vacancies on the O anions. This substitution facilitated the generation of oxygen vacancies within CeO_2_ (as illustrated in Fig. 2d and 5d). These oxygen vacancies in oxidase mimic species are postulated to enhance the material's affinity for oxygen, which in turn amplifies its catalytic activity.^50^ Secondly, prior research has indicated that the modification with imidazole can alter the electronic state of Ce in Ce-based materials. This electronic state adjustment improves the material's affinity for oxygen, further contributing to the enhanced catalytic performance.^41^

**Scheme 2:**
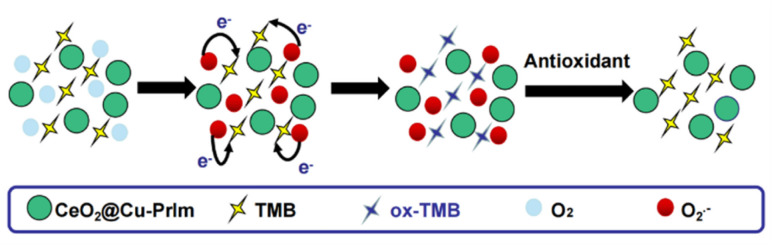
The potential reaction mechanisms for the reaction system of CeO_2_@Cu-PrIm/ox-TMB assay.

The potential reaction mechanisms were proposed (Scheme 2). Since CeO_2_@Cu-PrIm had multiple oxidation states Ce^3+^/Ce^4+^, Cu^+^/Cu^2+^, this was conducive to the metal ions transferring the electrons to O_2_ to form O_2_˙^−^. This strongly oxidizing intermediate transferred the electrons to TMB. Therefore TMB completed the transformation into ox-TMB. The detection of antioxidants could be accomplished through the difference in absorbance caused by electron transfer between antioxidants and ox-TMB.

### 3.5. Detection of the typical antioxidant

**Fig. 6:**
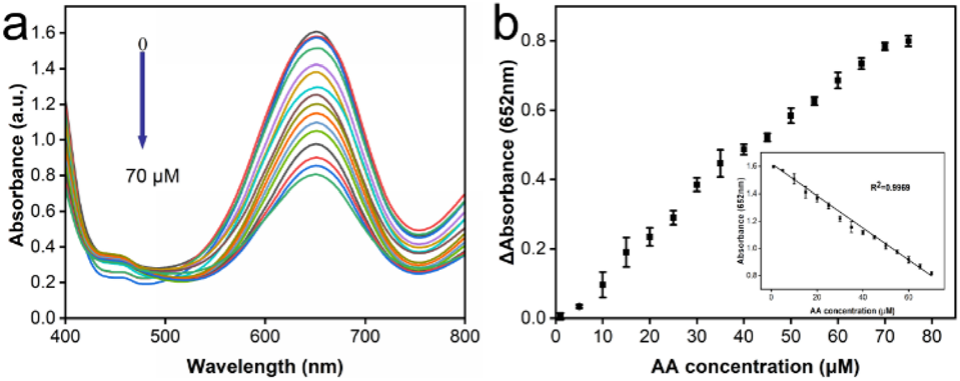
Detection of the typical antioxidant. (a) UV-vis absorbance spectra encompass concentrations ranging from 0 to 70 μM. (b) The alteration in absorbance at 652 nm with AA concentration and the inset the linear dependence of change in absorbance on the AA concentration from 1 to 70 μM.

To conduct the detection of antioxidants, ascorbic acid (AA) was selected as a representative of typical antioxidants for detection for the purpose of this study. Antioxidants could inhibit reactions based on the activity of oxidase-like enzymes, where the concentration of antioxidants could be deduced from the degree of inhibition. A colorimetric assay was carried out under the specified screened conditions (Fig. 6a). As AA was introduced into the reaction system, the characteristic peaks corresponding to the oxidized TMB exhibited a gradual reduction in intensity with increasing concentrations of AA. The relationship between the difference in absorbance at 652 nm from its initial value in relation to the varying AA concentration was illustrated and the inset highlights a strong inear correlation between the change in absorbance and AA concentration across the range of 1 to 70 μM (Fig. 6b). Further, the equation derived from the measured data, *y* = 1.62004–0.01174× (with an *R*^2^ value of 0.9969) described the detection of AA very well. The limit of detection (LOD) for this colorimetric AA detection method was determined to be 1.26 μM, calculated using the 3S/N formula as a basis.^31^ All these results provided vigorous evidence that the reaction system of the CeO_2_@Cu-PrIm/ox-TMB assay was capable of detecting the antioxidant with satisfactory sensitivity. Besides, our work had a better LOD and linear range when contrasted with certain other reported nanozymes. This observation reinforces the notion that our method was poised to excel as a well-performing approach in TAC assays (Table S2†).

To further validate the accuracy of the reaction system of CeO_2_@Cu-PrIm/ox-TMB assay proposed in this study for colorimetric antioxidant detection, the DPPH˙ free radicals scavenging experiment with good performance in TAC detection was selected for the assessment. The results within the same AA concentration range (Fig. S5†) also demonstrated a good linear correlation (with an *R*^2^ value of 0.9903) and a LOD of 1.35 μM. In summary, our suggested CeO_2_@Cu-PrIm/ox-TMB-based approach proved to be dependable for the quantitative determination of antioxidants using colorimetric detection.

To explore the selectivity of the reaction system of CeO_2_@Cu-PrIm/ox-TMB assay for the detection of antioxidants, the interference ability of some potential substances that might interfere with the detection was evaluated by the interfering substances, including K^+^, Na^+^, Mg^2+^, Ca^2+^, Cl^−^, glucose, maltose, fructose, galactose, imidazole, 2-methylimidazole, 2-ethylimidazole, 2-propylimidazole and 2-buthylimidazole. Except for imidazole and 2-methylimidazole, various interferents produced some insignificant interference in the detection of antioxidants, demonstrating the selectivity of the assay in detecting antioxidants (Fig. S6†). In summary, the above results verified the potential of the reaction system of CeO_2_@Cu-PrIm/ox-TMB assay for TAC determination of real samples.

### 3.6. Detection of TAC of real samples

**Fig. 7:**
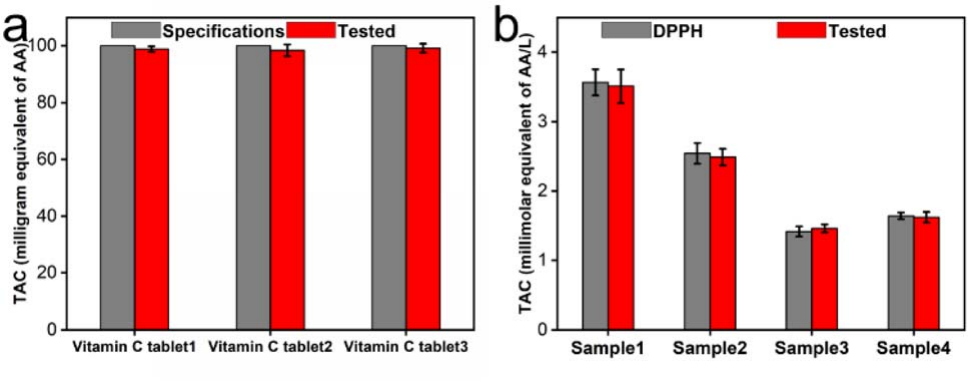
Detection of TAC using the proposed assay. (a) TAC detection of different vitamin C tablets, validating method accuracy by comparing against known vitamin C tablet specifications. (b) TAC detection of various samples, including kiwi fruit, orange, tomato, and orange juice.

To further validate the practicability of the reaction system of CeO_2_@Cu-PrIm/ox-TMB assay in TAC detection, we applied this method to several real samples of different vitamin C tablets and fruits. The TAC content of the different vitamin C tablets was initially assessed using the reaction system of CeO_2_@Cu-PrIm/ox-TMB assay and validated by the DPPH˙ measurement method. The TAC contents obtained by the two detection methods were closely aligned with the established AA standard specifications found in vitamin C tablets (Fig. 7a and S7†). Under identical conditions, the TAC contents of kiwi fruit, orange, tomato, and orange juice were evaluated, which were basically consistent with the values obtained by the DPPH˙ measurement method (Fig. 7b). These results demonstrated the practical applicability of the proposed reaction system of CeO_2_@Cu-PrIm/ox-TMB assay relative to the TAC detection of real samples such as fruits.

## 4. Conclusions

In conclusion, the CeO_2_@Cu-PrIm obtained by our simple synthetic method was to be an oxidase-like nanozyme for TAC detection. CeO_2_@Cu-PrIm had oxidation states of Ce^3+^/Ce^4+^, Cu^+^/Cu^2+^. Notably, the presence of a multi-state cycle facilitated the efficient electron transfer from metal ions to O2 to form O2˙^−^. Based on the proposed mechanism, a TAC detection method represented by AA was proposed in this study, The colorimetric sensor exhibited a linear response over a concentration range of 1 μM to 70 μM, accompanied by a limit of detection (LOD) of 1.26 μM The accuracy and selectivity of the reaction system of CeO_2_@Cu-PrIm/ox-TMB assay in real samples were investigated *via* anti-interference experiments, real samples, with reference to the corresponding specifications or the results of the DPPH˙ radical assay method. This proposed detection method was assessing TAC within real samples, such as fruits, which showed great potential for expansion into analytical assay applications.

## Data availability

The authors declare that the data supporting the findings of this study are available within the paper and its ESI† files. Should any raw data files be needed in another format they are available from the corresponding author upon reasonable request. Source data are provided with this paper.

## Author contributions

Zhendong Fu: writing – original draft, methodology, formal analysis. Jiahe Qiu: writing – original draft, methodology, formal analysis. Ping Gong: conceptualization, supervision. Danhong zhang: conceptualization, supervision. Liping Wang: conceptualization, writing – review & editing, supervision.

## Conflicts of interest

There are no conflicts to declare.

## Supplementary Material

RA-015-D4RA07858F-s001
