# Supplementary material for: Cu-doped and 2-propylimidazole-modified nanoceria (CeO2@Cu-PrIm) oxidase-like nanozyme for total antioxidant capacity assay of fruits
Source: RSC Adv. 2025 Apr 1;15(13):9997–10004. doi: 10.1039/d4ra07858f (PMC11959357; doi:10.1039/d4ra07858f)
Supplement: RA-015-D4RA07858F-s001 [file RA-015-D4RA07858F-s001.pdf]

Supplementary Information (SI) for RSC Advances.
This journal is © The Royal Society of Chemistry 2025

Supporting Information

Cu-doped and 2-propylimidazole-modified nanoceria (CeO_2@Cu-PrIm) oxidase-like nanozyme for total antioxidant capacity assay of fruits

Zhendong Fu[a1], Jiahe Qiu[a1], Ping Gong[a], Danhong Zhang[b*], . Liping Wang[a*]

[a] Key Laboratory for Molecular Enzymology and Engineering of Ministry of Education, School of Life Sciences, Jilin University, Changchun 130012, China

[b] Jilin University Hospital, Jilin University, Changchun 130012, China

[1] These authors contributed equally to this work and should be considered as co-first authors

[*] Correspondence: wanglp@jlu.edu.cn; Zhangdanhong@jlu.edu.cn; Tel.: +86-431-8515-5348

Jiahe Qiu qiujh1319@mails.jlu.edu.cn

Zhendong Fu fuzd21@mails.jlu.edu.cn

Ping Gong ping19962020@163.com

Danhong Zhang zhangdanhong@jlu.edu.cn

Liping Wang wanglp@jlu.edu.cn

■ **Supplementary Figures**

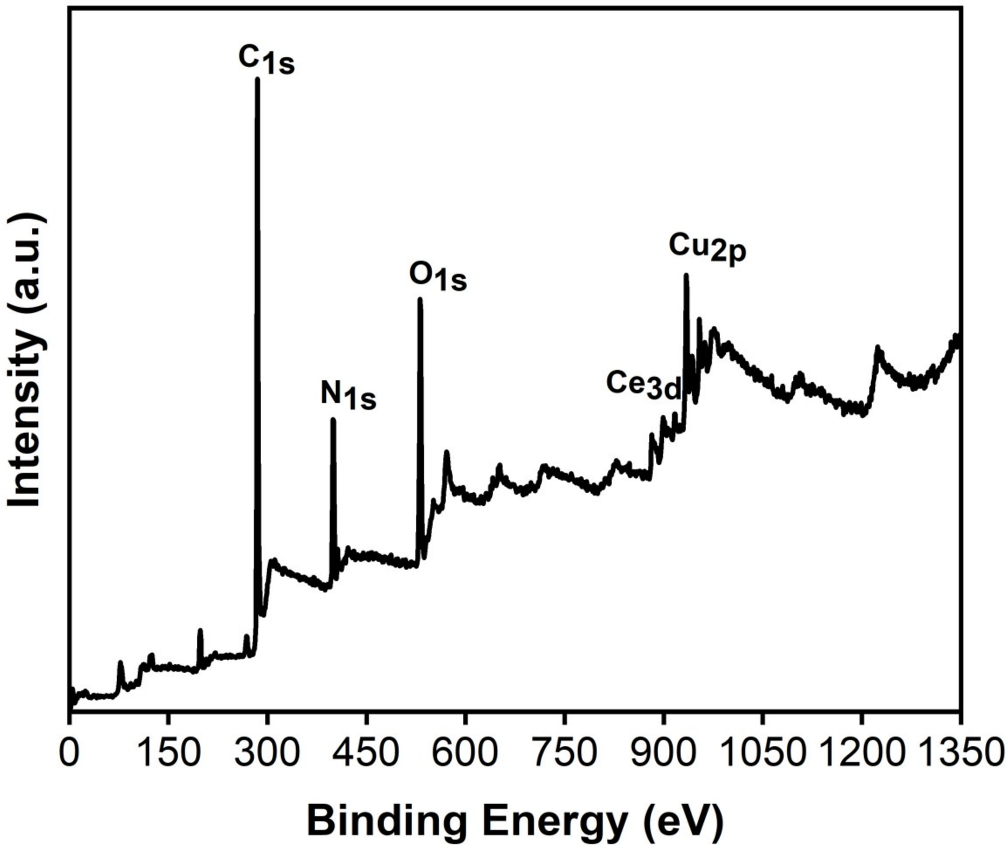

Fig. S1. XPS spectra of survey scan for CeO_2@Cu-PrIm.

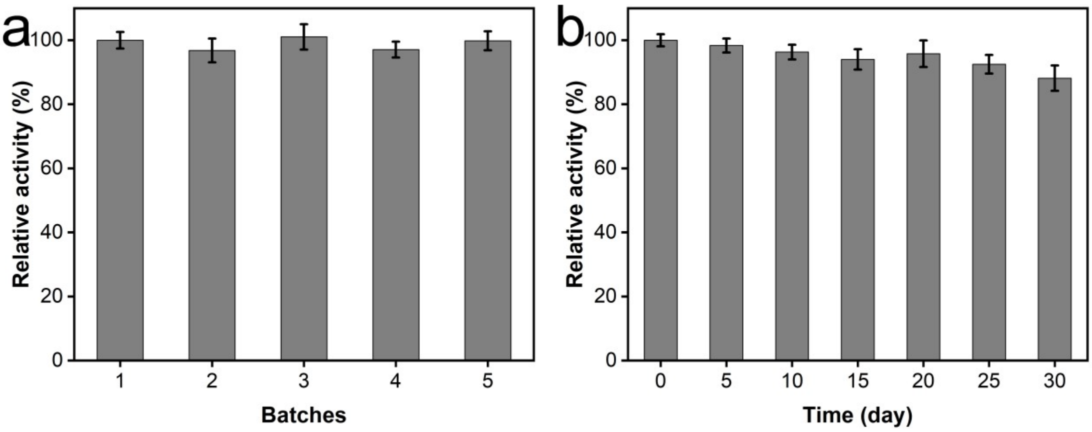

Fig. S2. The stability of the oxidase-like catalytic activity of CeO_2@Cu-PrIm. (a) Batch stability of CeO_2@Cu-PrIm. (b) Storage stability of CeO_2@Cu-PrIm.

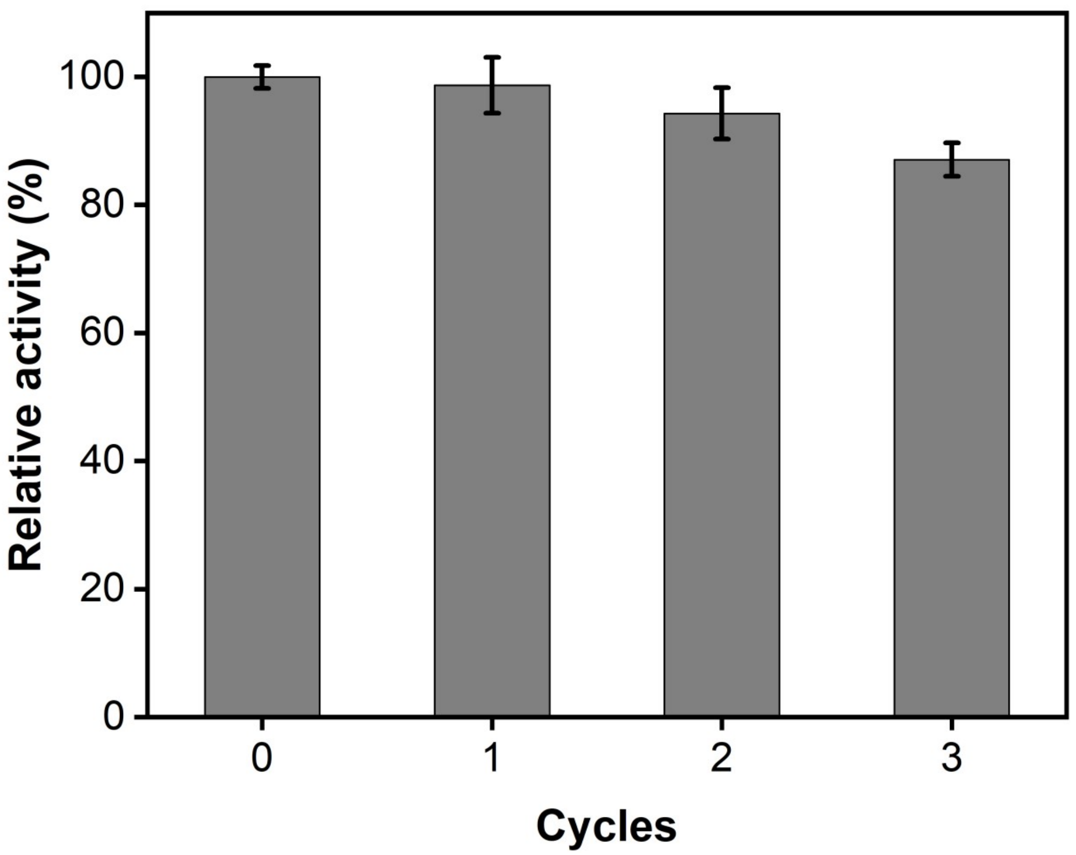

Fig. S3. The oxidase-like activity of CeO_2@Cu-PrIm after the cycle of centrifugation. The recycled CeO_2@Cu-PrIm was obtained by centrifuging the reaction solution at 8000 g for 10 min. The above cycle was repeated five times.

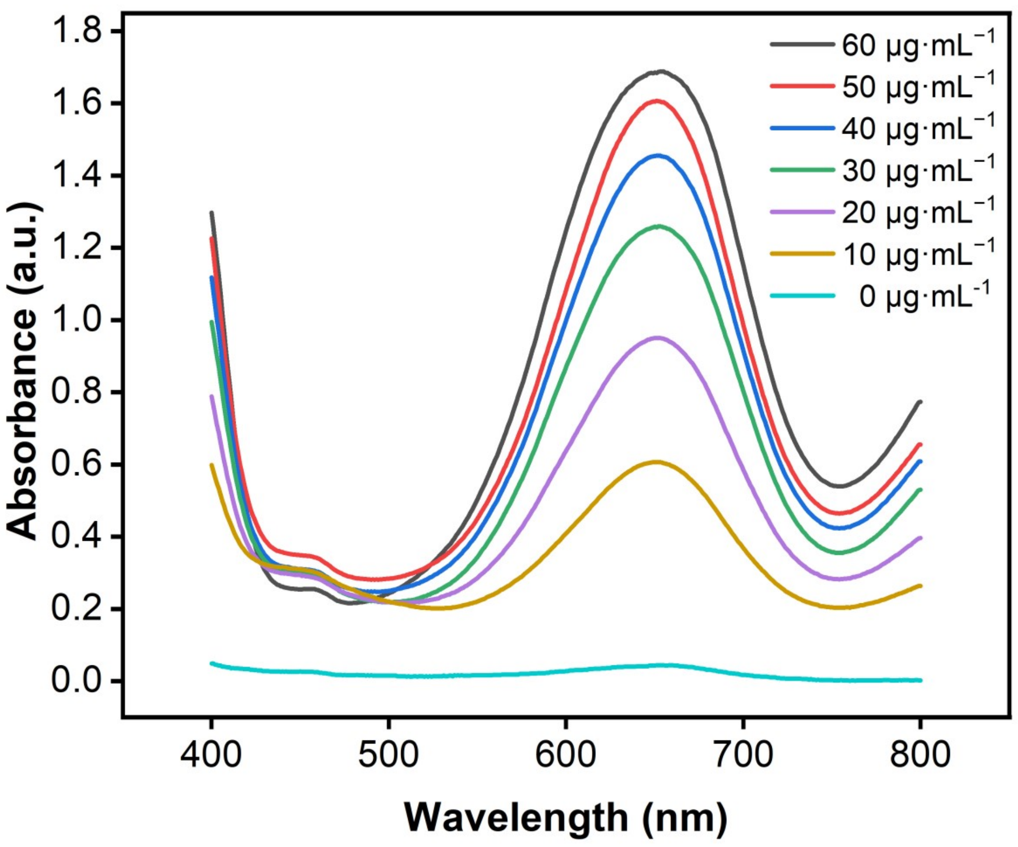

Fig. S4. The UV-vis absorption spectra describing the relationship between CeO_2@Cu-PrIm catalytic activity and concentration.

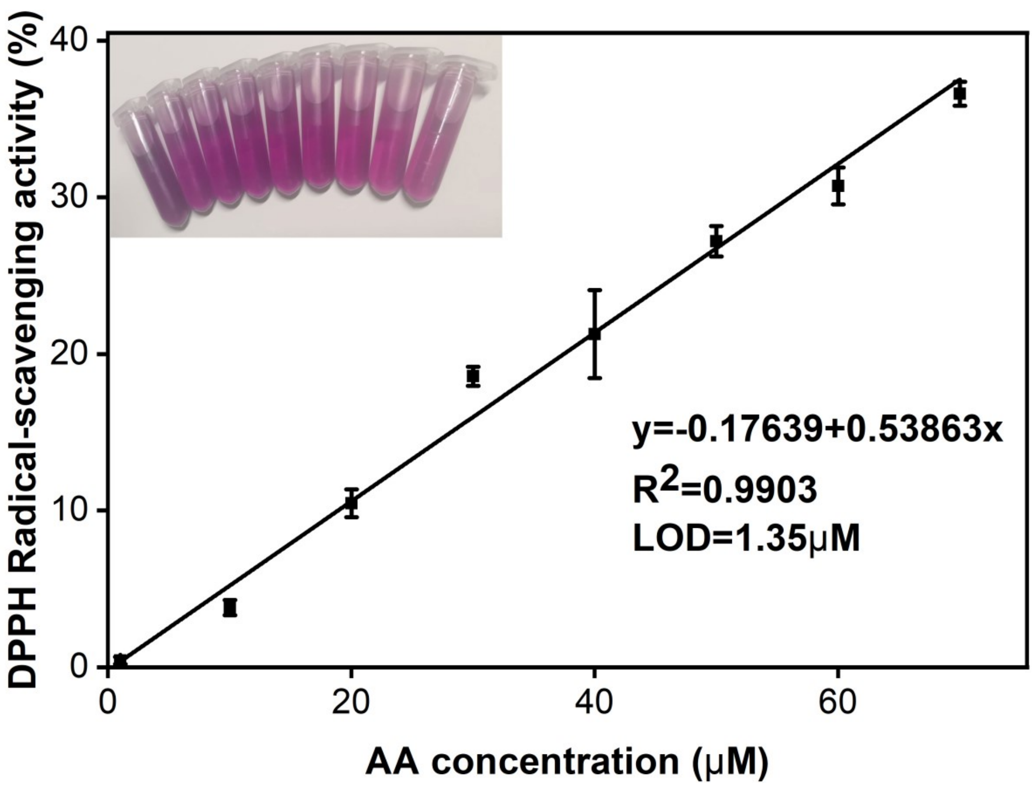

Fig. S5. The detection of AA using the DPPH· free radical scavenging method.

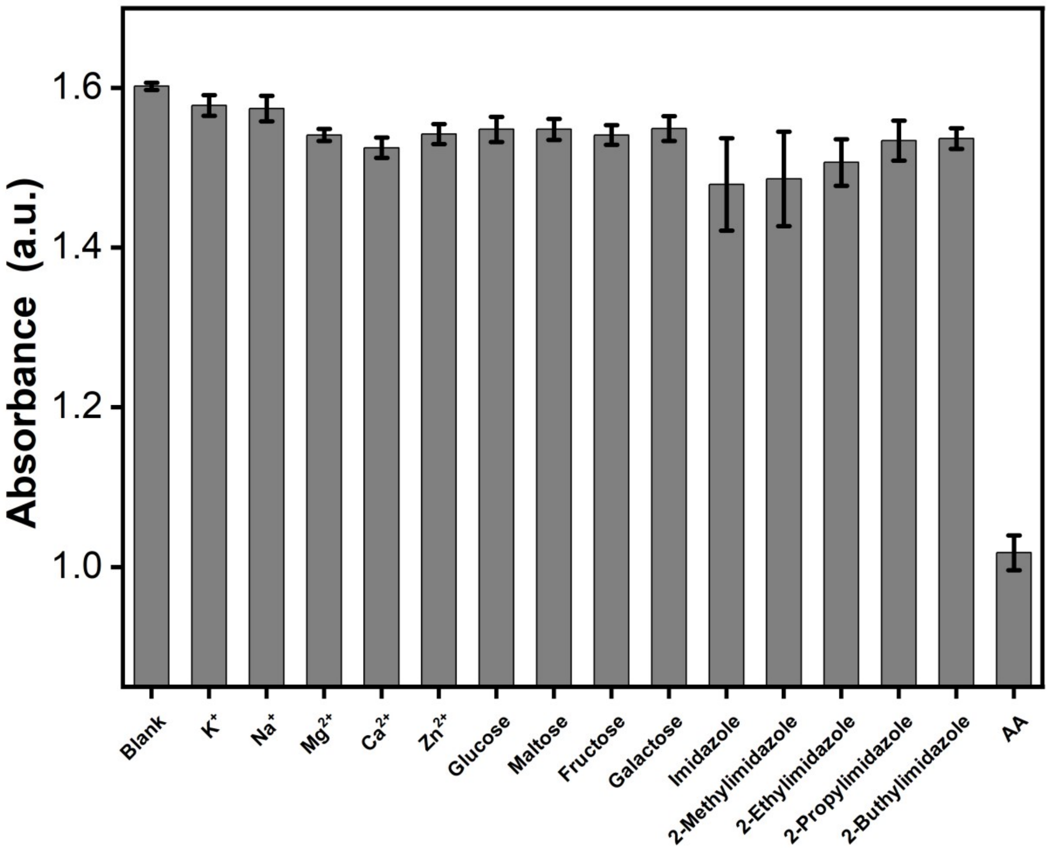

Fig. S6. The anti-interference capacity of the reaction system of CeO_2@Cu-PrIm/ox-TMB assay.

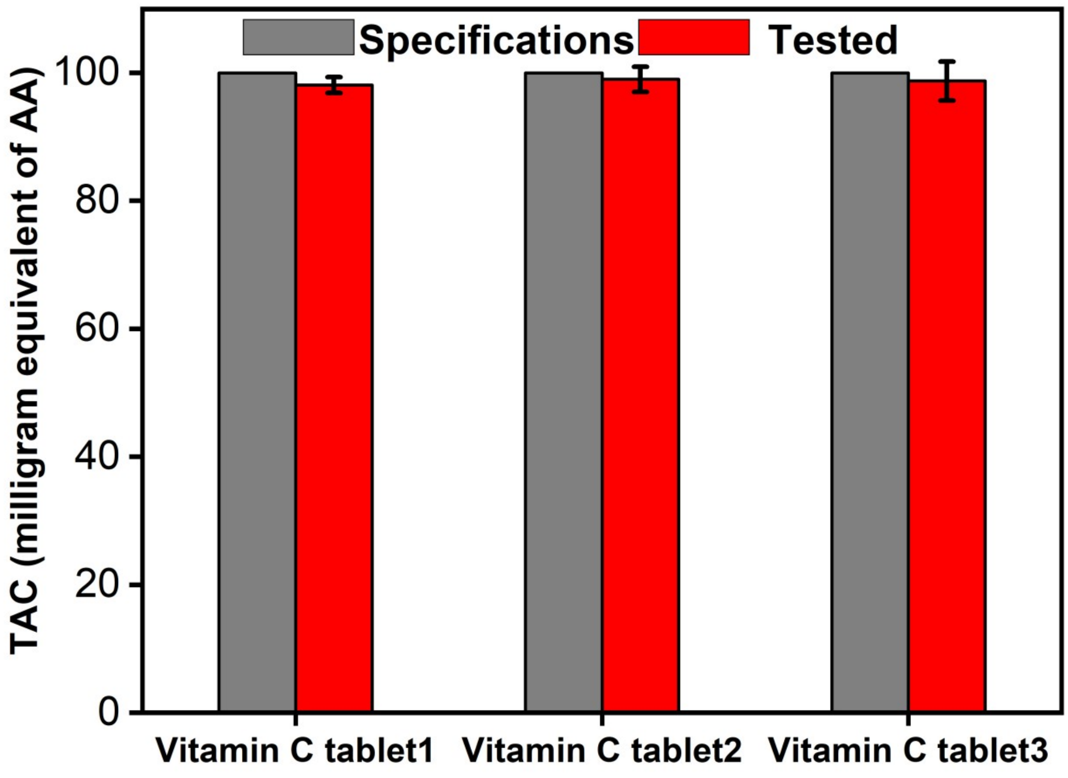

Fig. S7. The detection of TAC of three vitamin C tablets using the DPPH· free radical scavenging method.

Supplementary Tables

Table S1. Comparison of kinetic parameters between CeO_2@Cu-PrIm and reported oxidase mimics.

Catalyst	K_m [mM]	V_{max} [10^{-8} M/s]	Reference
CeO_2@Cu-PrIm	6.521	77.45	This work
CeO_2 NPs	3.8	70	[1]
Nano-CeO_2	0.42	10.04	[2]
Ce-BPyDC	0.16	26.8	[3]
Ce-MOF (MVCM)	0.00037	550	[4]
Dex-FeMnzyme	0.33	13.29	[5]
MIL-53(Fe)	1.08	8.78	[6]
Mn_3O_4 NPs	0.025	5.07	[7]

Table S2. Comparison of reported oxidase mimics for the detection of AA.

Nanomaterials	Linear range (μM)	LOD (μM)	Reference
CeO_2@Cu-PrIm	1-70	1.26	This work
Ce-BPyDC	1-20	0.28	[3]
Dex-FeMnzyme	1-30	1.17	[5]
MIL-53(Fe)	28.6-190.5	15	[6]
CuNCs	0.5-10	0.11	[8]
CP_{600-6}	0.8-80	35	[9]
SNC	100-5000	80	[10]
Fe-NC NTs	0.2-20	0.131	[11]

Reference

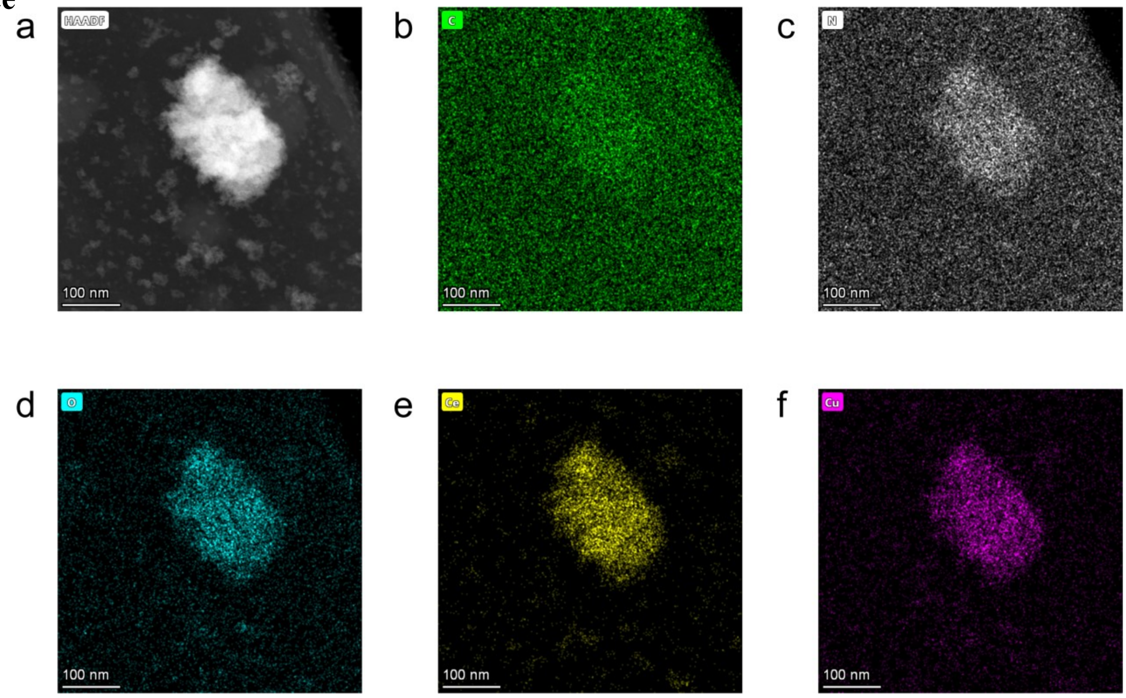

2025-01-15 18:48:16 Analysis of spectrum: Spectra from Area #1

Z	Element	Family	Atomic Fraction (%)	Atomic Error (%)	Mass Fraction (%)	Mass Error (%)	Fit error (%)
6	C	K	74.64	6.23	42.83	2.44	3.31
7	N	K	0.74	0.28	0.50	0.19	31.43
8	O	K	14.77	3.18	11.29	2.33	2.33
17	Cl	K	0.43	0.09	0.74	0.14	3.17
29	Cu	K	5.00	0.77	15.19	2.14	0.14
58	Ce	L	4.40	0.58	29.45	3.47	0.21

Fig. S8. The EDS Mapping of CeO_2@Cu-PrIm NPs (a) HAADF of CeO_2@Cu-PrIm NPs (b) C atoms (c) N atoms (d) O atoms (e) Ce atoms (f) Cu atoms

1. A. Asati, S. Santra, C. Kaittanis, S. Nath and J. M. Perez, *Angewandte Chemie International Edition*, 2009, **48**, 2308-2312.
2. H. Cheng, S. Lin, F. Muhammad, Y.-W. Lin and H. Wei, *ACS Sensors*, 2016, **1**, 1336-1343.
3. L. Luo, L. Huang, X. Liu, W. Zhang, X. Yao, L. Dou, X. Zhang, Y. Nian, J. Sun and J. Wang, *Inorganic Chemistry*, 2019, **58**, 11382-11388.
4. Y. Xiong, S. Chen, F. Ye, L. Su, C. Zhang, S. Shen and S. Zhao, *Chemical Communications*, 2015, **51**, 4635-4638.
5. X. Han, L. Liu, H. Gong, L. Luo, Y. Han, J. Fan, C. Xu, T. Yue, J. Wang and W. Zhang, *Food Chemistry*, 2022, **371**, 131115.
6. L. Ai, L. Li, C. Zhang, J. Fu and J. Jiang, *Chemistry – A European Journal*, 2013, **19**, 15105-15108.
7. X. Zhang and Y. Huang, *Analytical Methods*, 2015, **7**, 8640-8646.
8. H. Rao, H. Ge, Z. Lu, W. Liu, Z. Chen, Z. Zhang, X. Wang, P. Zou, Y. Wang, H. He and X. Zeng, *Microchimica Acta*, 2016, **183**, 1651-1657.
9. Z. Lou, S. Zhao, Q. Wang and H. Wei, *Analytical Chemistry*, 2019, **91**, 15267-15274.
10. Y. Chen, L. Jiao, H. Yan, W. Xu, Y. Wu, H. Wang, W. Gu and C. Zhu, *Analytical Chemistry*, 2020, **92**, 13518-13524.
11. N. Song, M. Zhong, J. Xu, C. Wang and X. Lu, *Sensors and Actuators B: Chemical*, 2022, **351**, 130969.
